# The Use of Vaping Among Male Health Sciences Students Compared to Other Male Students in Riyadh

**DOI:** 10.7759/cureus.51257

**Published:** 2023-12-28

**Authors:** Abdulrahman Alhaqbani, Mohammed Alismail, Anas Alotaibi, Ziyad Alibrahim, Abdulhadi Alqahtani, Aamir Omair, Sami Al-Nasser

**Affiliations:** 1 College of Medicine, King Saud Bin Abdulaziz University for Health Sciences, Riyadh, SAU; 2 College of Medicine, King Abdullah International Medical Research Center, Riyadh, SAU; 3 Department of Medical Education, College of Medicine, King Saud Bin Abdulaziz University for Health Sciences, Riyadh, SAU

**Keywords:** riyadh, university students, conventional smoking, e-cigarettes, vaping

## Abstract

Background

Vaping has become widely used by teenagers due to its accessibility, variety of flavors, peer influence, and the thought that it is a less harmful alternative to tobacco smoking. This study aimed to compare the prevalence of vaping among health sciences students compared to other college students in Riyadh and identify reasons for its usage.

Methods

A cross-sectional study was conducted in three major universities of Riyadh: King Saud bin Abdulaziz University, King Saud bin Abdulaziz University for Health Sciences, and Al-Imam Mohammad Ibn Saud Islamic University. A self-administered online questionnaire related to the use of e-cigarettes and conventional cigarettes was utilized. It included questions about the use of conventional cigarettes and e-cigarettes and the reasons for using them. Responses were compared between health sciences and non-health sciences students in Riyadh.

Results

An electronic survey was distributed online, and 442 students responded, but two of them did not agree to participate, so they were removed from the sample. Out of 440 students, 312 (71%) were health sciences students, and 128 (29%) were non-health sciences students. Smoking conventional cigarettes was found among 38 (12%) health sciences students, and 22 (17%) non-health college students smoked conventional cigarettes (p=0.16). Regarding vaping, 117 (38%) health sciences students smoked e-cigarettes. On the other hand, 47 (39%) non-health college students smoked e-cigarettes (p=0.99). Anxiety/stress relief (54%) and peer influence (46%) were the most common reasons for those who smoked conventional cigarettes. Regarding the most common reasons behind using e-cigarettes, the majority (55%) considered e-cigarettes less harmful than conventional cigarettes. The second most common reason was having no distinctive odor (36%).

Conclusion

The study found that there was a high prevalence of the self-reported use of e-cigarettes. It appears that the use of conventional cigarette smoking is not as common as e-cigarettes among university students. This study found that university students tend to use e-cigarettes more than conventional cigarettes due to the belief that e-cigarettes are less harmful than conventional cigarettes.

## Introduction

Electronic cigarette inhalation also known as ‘vaping’ is an alternative to conventional smoking. It is a mixture of chemicals in the form of liquids that come in many different flavors with optional nicotine that will be turned into vapor and inhaled [1]. Electronic cigarettes have gradually become more popular as an alternative, specifically among teenagers, to conventional smoking, through burning, giving many harmful and carcinogenic results [2]. However, vaporizers utilize a mixture of liquids often made from vegetable glycerin or propylene glycol mixed with different additives such as nicotine and flavors. The usage of electronic cigarettes increased by approximately four times between 2009 and 2010 [3]. Vaping devices marked an increase in the consumption rate in the conventional smoking population [4].

Many studies have discussed the prevalence and epidemiology of e-cigarettes. For instance, 11% of 1259 university students smoked e-cigarettes in a study that was conducted in Jordan [5]. Moreover, a study in Qatar found that 16% of male students out of 199 university students smoked e-cigarettes [6]. While in Saudi Arabia, a study conducted in Jeddah on a sample of 1007 students from four different health colleges showed that more than one in four was a user of e-cigarettes [7]. Furthermore, a cross-sectional study from King Saud University in Riyadh, Saudi Arabia, on 480 university students showed that 26% used e-cigarettes [8].

People tend to use e-cigarettes for many different reasons, including accessibility, flavors, relatives/friends' impact, interest, and the idea that vaporizers are revolutionized and better substitutes for conventional tobacco smoke [9]. A cross-sectional study conducted at Qassim University in Buraydah showed that approximately 1 in 10 students confirmed that they had smoked an e-cigarette, and approximately 23% of those surveyed believed that e-cigarettes could help with the prevention of smoking while only 11% said they would recommend it to a patient; however, 50% agreed that e-cigarettes are addictive [10]. In addition, an electronic questionnaire was distributed to 900 students from one private medical college and two public government medical colleges in Riyadh, Saudi Arabia. A total of 636 students responded to the questionnaire, and 3% of the participants stated that they used electronic cigarettes while 6% of the respondents answered they had tried electronic cigarettes before [11]. Moreover, a cross-sectional study was conducted at Al-Faisal University in Riyadh on 401 medical students using a questionnaire, and the results showed that 12% of those students were using vaping devices [12]. Currently, current smokers and previous smokers use vaping devices as a remedy to replace nicotine and to quit or at least reduce smoking [13]. On the other hand, some individuals use vaping devices as a less harmful option compared to conventional smoking [14]. Vaping devices have contributed to the decrease in the usage of conventional cigarettes by approximately 80% [15]. Despite the use of vaping devices to aid in the cessation of conventional smoking, the US FDA (Food and Drug Administration) has no approval for such devices [16].

There are many short-term complications, which might lead to death. According to the Centers for Disease Control and Prevention (CDC), 2807 cases or deaths were reported from 50 states in the USA as a result of e-cigarettes, or vaping, product use-associated lung injury (EVALI) [17]. However, there are no studies that indicate a long-term effect because when compared to other tobacco products, e-cigarettes are considered a new method.

The prevalence of vaping has been on the rise globally, and it is important to investigate its impact on specific populations such as male health sciences students. These students are future healthcare practitioners and their behaviors can influence patient counseling and healthcare practices. Understanding vaping patterns among this group is crucial for devising targeted interventions and policies to reduce vaping-related harm. Also, while there have been studies on vaping among general student populations, there is a scarcity of research specifically focusing on male health sciences students. This study aims to fill this gap in the literature by providing insights into the prevalence, patterns, and factors associated with vaping among this specific group.

Furthermore, Riyadh, the capital city of Saudi Arabia, has unique social, cultural, and environmental factors that may influence vaping behaviors. Investigating vaping among male health sciences students in Riyadh can provide localized data to inform public health strategies and interventions tailored to the local context. Therefore, this research aimed to fill a gap in the knowledge and understanding of how this new trend is spreading among university students and the reasons for its popularity. The primary objective of this research was to determine the use of vaping among male health sciences students compared to other male students in Riyadh. Our hypothesis for this study was that male health sciences students in Riyadh would have a higher prevalence of vaping compared to other male students. We predicted that the specific academic and professional environment of health sciences students, coupled with potential stressors and social influences, might contribute to increased rates of vaping within this subgroup.

## Materials and methods

This study was conducted on male students in Riyadh’s three major universities, which were King Saud bin Abdulaziz University for Health Sciences (KSAU-HS), King Saud University (KSU), and Imam Muhammad Ibn Saud Islamic University (IMSIU). KSAU-HS is the first public university in the Kingdom of Saudi Arabia and the Middle East region that specializes in health sciences (approximately 2,500 male students in Riyadh). KSU was founded in 1957 by King Saud bin Abdulaziz as Riyadh University (approximately 38,500 students). IMSIU was founded in 1953 (approximately 30,000 students). Responses from some private universities were accepted, including Riyadh Elm University, AlMaarefa University, Prince Sultan University (PSU), and Al Yamamah University.

This cross-sectional study was conducted between two groups: health and non-health sciences students. The population included male students from health and non-health sciences colleges in KSAU-HS, KSU, IMSIU, and private universities (Saudis & non-Saudis). The total approximate number of students is approximately 71,000.

For a population of approximately 71,000 students and a significance level of 5% with a power of 80%, the sample size that was calculated by using the OpenEpi sample size calculator (https://www.openepi.com/SampleSize/SSCohort.htm) was 383. Since there were two populations, the total sample size was 766 divided into health sciences students (n=383) and non-health sciences students (n=383). A non-probability convenience sampling technique was used by distributing an online questionnaire to students.

After obtaining permission from the original authors, a self-reported questionnaire that was related to vaping and classical smoking was used [7]. The questionnaire consisted of 18 questions in English. The construction of the questionnaire was validated by an expert methodologist, and the content of the questionnaire was validated by a specialist pulmonologist [7]. The questions in this questionnaire were classified into several categories, such as vaping-related questions (reason for consumption, variety of flavors, frequency, smoking condition) and sociodemographic data (major, age) [7].

The main outcome variable was to identify the use of e-cigarettes, the grouping variables were health and non-health science students. SPSS version 22 was used for data entry and analysis. Categorical variables, such as users of e-cigarettes, non-users, and health and non-health sciences students, were reported as percentages and frequencies. The chi-square test was used to find the association between the aforementioned categorical variables. A test was considered significant if the p-value was less than 0.05.

No information from which a participant could be identified was collected. All participants were provided with informed consent.

## Results

An electronic survey was distributed online; 442 students responded but two of them did not agree to participate so they were removed from the sample. Out of the 440 respondents, 398 (90%) were from Riyadh’s major universities (KSAUH, KSU, IMSIU) while 42 (10%) were from private universities in Riyadh. Out of 440 students, 312 (71%) were from health sciences colleges, whereas 128 (29%) were from non-health sciences colleges (Table 1).

**Table 1:** General characteristics of the respondents (N=440)

		n	%
What is your university?	King Saud bin Abdulaziz University for Health Sciences (KSAU-HS).	119	27%
	King Saud bin Abdulaziz University (KSU)	207	47%
	Al-Imam Mohammad Ibn Saud Islamic University	72	16%
	Others	42	10%
Health sciences vs non-health colleges	Health Sciences	312	71%
	Non-Health College	128	29%
What is your specialty?	College of Medicine (COM)	192	44%
	College of Applied Medical Sciences (CAMS)	47	11%
	College of Pharmacy (COP)	45	10%
	College of Dentistry (COD)	28	6%
	Other	128	29%
Smoke cigarettes	Yes	60	14%
	No	378	86%
Have you ever tried an electronic cigarette?	Yes	164	38%
	No	262	62%

**Figure 1:**
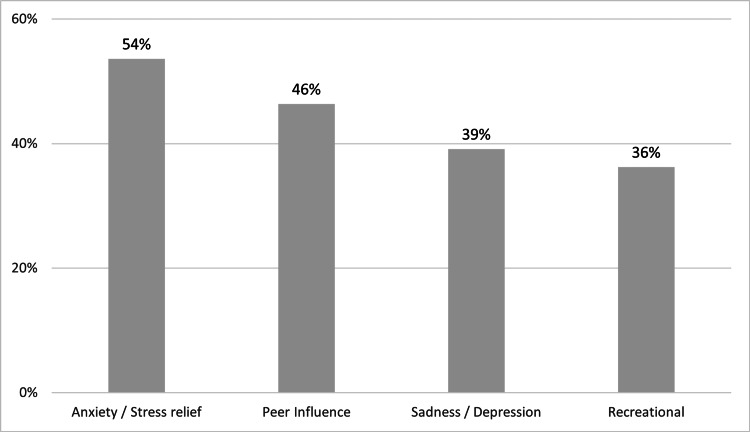
Reasons for smoking conventional cigarettes (N=60)

Anxiety/stress relief (54%) and peer influence (46%) were the most common reasons for those who smoked conventional cigarettes (Figure 1). 

**Figure 2:**
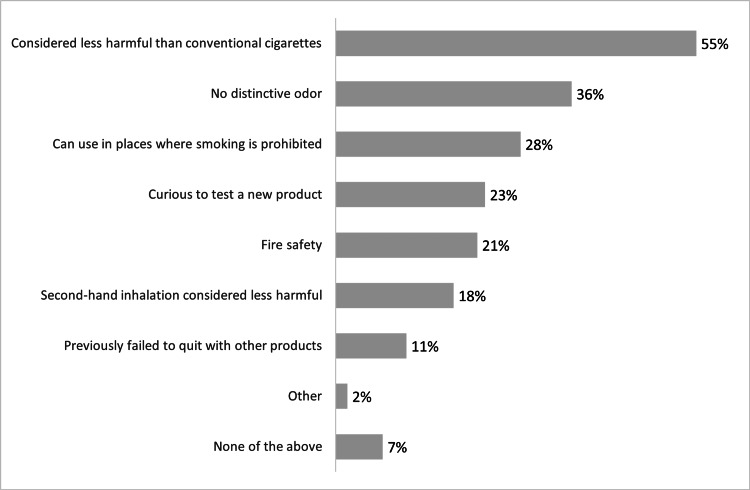
Reasons for smoking electronic cigarettes (N=164)

The 164 respondents who smoked e-cigarettes were asked about what attracted them to e-cigarettes. The majority (55%) considered e-cigarettes less harmful than conventional cigarettes as the most common reason behind using e-cigarettes. The second most common reason was having no distinctive odor (36%), and the ability to use it in places where conventional smoking is prohibited (28%) was the third most common reason (Figure 2).

The use of conventional and e-cigarettes was compared between the health sciences and non-health sciences students as shown in Table 2. Regarding students who smoked conventional cigarettes, 38 (12%) were from health sciences colleges and 22 (17%) were from non-health colleges (p=0.16). Among students who smoked e-cigarettes, 117 (38%) were health sciences students and 47 (39%) were students from non-health colleges (p=0.99).

**Table 2:** Comparing the use of conventional and e-cigarettes between health sciences and non-health sciences students

		Health Sciences vs non-Health Colleges				
		Health Science		Non-Health College		
		n	%	n	%	p-value
Do you smoke conventional (classical) cigarettes?	Yes	38	12%	22	17%	0.16
	No	273	88%	105	83%	
Have you ever tried an electronic cigarette (even 1 or 2 puffs)?	Yes	117	38%	47	39%	0.99
	No	187	62%	75	61%	

## Discussion

This study compared the prevalence, reasons, and frequency of e-cigarette and conventional cigarette use between health science students and non-health science students. In the current study, almost two out of five students stated that they had tried e-cigarettes at least once in their lifetime compared to one out of seven using conventional cigarettes. The study found that e-cigarettes are more popular than conventional cigarettes among university students, which is also found in the results obtained in a similar study in Jeddah [7]. This seems to be mainly due to the belief that e-cigarettes are less harmful than conventional cigarettes.

No significant difference in the use of e-cigarettes was seen between health students and non-health sciences students. Furthermore, the prevalence of e-cigarette users in this study (38%) is much higher than that in other studies in the region. In 2019, a study conducted in Jeddah had a prevalence of 28% of students who used e-cigarettes [7]. Moreover, a study in 2021 that included university students of both genders in Jordan had a result of approximately 11% using e-cigarettes [5]. This difference might be caused by the exclusion of females in the current study. In 2021, a study among Qatar University students found a prevalence of 16% in male students who smoked e-cigarettes [6]. The prevalence of e-cigarette users among university students in Saudi Arabia is higher than that among university students in Jordan and Qatar [5-7]. These are worrying numbers, as the long-term effects of e-cigarettes are not well-known, but evidence shows short-term effects and complications such as e-cigarette or vaping use-associated lung injury (EVALI) [18].

In this study, almost one-fourth of the respondents stated they would recommend e-cigarettes as a good way to quit conventional smoking; however, only one-third of e-cigarette users claimed that they were able to quit conventional smoking. In 2017, a study in the United States found that e-cigarettes serve as a gateway to conventional cigarette smoking initiation [19]. This is problematic because more than 85% of our study do not smoke conventional cigarettes, so the use of e-cigarettes can lead to conventional smoking and eventually addiction.

In the current study, the most common reason for e-cigarette use was the belief that e-cigarettes are less harmful than conventional cigarettes. This was followed by having no distinctive odor as the second most common reason, and the ability to use them in places where conventional smoking is prohibited was the third most common reason. A study in Jeddah revealed that the most common reason is entertainment, followed by peer effects [7]. Moreover, a study at Umm Al-Qura University showed that the most common reason for smoking e-cigarettes was the taste, followed by trying to quit smoking [20].

 In this study, it was found that smoking conventional cigarettes was not common in both health sciences and non-health sciences students, with a prevalence of 14%. In comparison with a study performed in 2011 in medical colleges at King Abdulaziz University, Jeddah, the prevalence of conventional cigarette users was 25% among male college students [21]. In Abha, Saudi Arabia, a study that was performed in 2018 showed that conventional smoking had a prevalence of 19% among male college students [22]. Furthermore, a study performed in 2016 among male students of Majmaah University, Saudi Arabia, showed an even higher prevalence of 30% conventional smokers among male college students [23].

Of those who smoked conventional cigarettes, the current study found that the most common reason students smoked conventional cigarettes was stress relief and anxiety, followed by peer influence. In 2011, a study among medical students at King Fahad Medical City in Riyadh showed that the most common reason was leisure and recreation, and peer influence was the second most common reason [24].

The authors acknowledge the sample limitations, which were limited mainly to three major universities and might not reflect the entire population of college students in Riyadh. They also acknowledge that the data might be subject to both recall and social desirability biases. Due to the coronavirus disease 2019 (COVID-19) situation, the questionnaire was administered online. This might be the reason for the low sample of non-health sciences students compared to health sciences students. Adding a question about which age the smoker started his behavior could have added more information that would have helped identify the pattern of smoking. Lastly, the authors could not include females in the study due to poor response rates and contact difficulties.

Also, it is crucial to acknowledge these limitations and their potential impact on the generalizability and precision of our findings to ensure transparency and prevent any misinterpretation of the results. Furthermore, we employed appropriate statistical methods, such as adjusting for sample size imbalances or using robust statistical techniques when appropriate, to minimize the biases associated with these limitations.

While the deficiencies in sample size and the lack of equal sample size for the subgroup are acknowledged as limitations of our study, we believe that the insights gained from the available sample still contribute valuable information to the existing literature and provide a foundation for future research. The study's findings hold implications for practice and policy interventions targeting male health sciences students in Riyadh, aiming to reduce vaping prevalence and mitigate potential health risks associated with vaping. The study's findings can inform the development of targeted intervention programs aimed at reducing vaping prevalence among male health sciences students in Riyadh. These programs can focus on raising awareness about the potential health risks associated with vaping, promoting healthier alternatives, and providing evidence-based cessation support tailored to the specific needs and challenges faced by this subgroup. Also, integrating comprehensive education on the risks and consequences of vaping into the curricula of health sciences programs can help equip future healthcare practitioners with the knowledge and skills necessary to address vaping-related issues in their practice. This can include training on effective communication strategies to counsel patients about vaping cessation and prevention.

Educational institutions can consider adopting or strengthening policies that prohibit vaping on campus premises, establish designated smoking/vaping areas, and enforce strict penalties for non-compliance. Such policies can create a supportive environment for male health sciences students and other students to abstain from vaping or seek assistance in quitting. Student health services can utilize the study's results to tailor their outreach efforts, provide targeted counseling services, and organize awareness campaigns specifically tailored to this demographic. This collaboration between academic institutions and student health services can enhance the effectiveness of prevention and cessation interventions.

Further research will be needed to explore the underlying factors contributing to higher vaping rates among male health sciences students. They can delve deeper into the specific motivations, attitudes, and social influences driving vaping behavior in this subgroup. Additionally, longitudinal studies can track the long-term health outcomes and changes in vaping patterns among male health sciences students to assess the effectiveness of interventions and policies implemented based on the current findings.

## Conclusions

The study found that there is a high prevalence of the use of e-cigarettes among university students. It appears that the use of conventional cigarette smoking is not common among university students as compared to e-cigarette smoking. The most common reason for e-cigarette use was the belief that e-cigarettes are less harmful than conventional cigarettes. The most common reasons students smoked conventional cigarettes are stress relief and anxiety, followed by peer influence. Further research is needed to gather more information on the patterns and causes of use and the effects of e-cigarettes on physical and mental health. Further studies comparing the use between males and females would help determine the prevalence and compare the reasons behind the use of vaping devices between males and females.
